# TSC patient-derived isogenic neural progenitor cells reveal altered early neurodevelopmental phenotypes and rapamycin-induced MNK-eIF4E signaling

**DOI:** 10.1186/s13229-019-0311-3

**Published:** 2020-01-06

**Authors:** Pauline Martin, Vilas Wagh, Surya A. Reis, Serkan Erdin, Roberta L. Beauchamp, Ghalib Shaikh, Michael Talkowski, Elizabeth Thiele, Steven D. Sheridan, Stephen J. Haggarty, Vijaya Ramesh

**Affiliations:** 1 Center for Genomic Medicine, Massachusetts General Hospital, Boston, MA 02114 USA; 2 MERCK Research Laboratories, Boston, MA 02115 USA; 3 Department of Neurology, Massachusetts General Hospital, 185 Cambridge Street, Boston, MA 02114 USA; 4 Center for Quantitative Health, Massachusetts General Hospital, Boston, MA 02114 USA

**Keywords:** Tuberous sclerosis complex, TSC1, mTORC1, Induced pluripotent stem cells, Neural progenitor cells, Early neurodevelopment, Disease modeling, CRISPR/Cas9, MEK-ERK1/2, MNK1/2-eIF4E

## Abstract

**Background:**

Tuberous sclerosis complex (TSC) is a neurodevelopmental disorder with frequent occurrence of epilepsy, autism spectrum disorder (ASD), intellectual disability (ID), and tumors in multiple organs. The aberrant activation of mTORC1 in TSC has led to treatment with mTORC1 inhibitor rapamycin as a lifelong therapy for tumors, but TSC-associated neurocognitive manifestations remain unaffected by rapamycin.

**Methods:**

Here, we generated patient-specific, induced pluripotent stem cells (iPSCs) from a TSC patient with a heterozygous, germline, nonsense mutation in exon 15 of *TSC1* and established an isogenic set of heterozygous (Het), null and corrected wildtype (Corr-WT) iPSCs using CRISPR/Cas9-mediated gene editing. We differentiated these iPSCs into neural progenitor cells (NPCs) and examined neurodevelopmental phenotypes, signaling and changes in gene expression by RNA-seq.

**Results:**

Differentiated NPCs revealed enlarged cell size in TSC1-Het and Null NPCs, consistent with mTORC1 activation. TSC1-Het and Null NPCs also revealed enhanced proliferation and altered neurite outgrowth in a genotype-dependent manner, which was not reversed by rapamycin. Transcriptome analyses of TSC1-NPCs revealed differentially expressed genes that display a genotype-dependent linear response, i.e., genes upregulated/downregulated in Het were further increased/decreased in Null. In particular, genes linked to ASD, epilepsy, and ID were significantly upregulated or downregulated warranting further investigation. In TSC1-Het and Null NPCs, we also observed basal activation of ERK1/2, which was further activated upon rapamycin treatment. Rapamycin also increased MNK1/2-eIF4E signaling in TSC1-deficient NPCs.

**Conclusion:**

MEK-ERK and MNK-eIF4E pathways regulate protein translation, and our results suggest that aberrant translation distinct in TSC1/2-deficient NPCs could play a role in neurodevelopmental defects. Our data showing upregulation of these signaling pathways by rapamycin support a strategy to combine a MEK or a MNK inhibitor with rapamycin that may be superior for TSC-associated CNS defects. Importantly, our generation of isogenic sets of NPCs from TSC patients provides a valuable platform for translatome and large-scale drug screening studies. Overall, our studies further support the notion that early developmental events such as NPC proliferation and initial process formation, such as neurite number and length that occur prior to neuronal differentiation, represent primary events in neurogenesis critical to disease pathogenesis of neurodevelopmental disorders such as ASD.

## Background

Tuberous sclerosis complex (TSC) is an autosomal dominant monogenic disorder with severe neurological manifestations including epilepsy, autism spectrum disorder (ASD), intellectual disability (ID), and hamartomas (benign tumor-like formations) in many organs. TSC is caused by mutations in the *TSC1* or *TSC2* gene, encoding tumor suppressor proteins hamartin (TSC1) and tuberin (TSC2) [1–3]. The TSC proteins form a functional complex that acts as a central hub relaying signals from diverse cellular pathways to inhibit mammalian/mechanistic target of rapamycin complex 1 (mTORC1) activity, which regulates cell growth and proliferation [4, 5]. In neuronal translation, mTORC1 signaling is a regulator of long-lasting synaptic plasticity and memory as it integrates signals from neuronal surface receptors/channels via MEK/ERK- and PI3K/AKT-mediated phosphorylation and inactivation of the TSC1-TSC2 complex [4–6]. The aberrant activation of mTORC1 in TSC has led to treatment with rapamycin analogs (rapalogs) as a lifelong therapy [7–10], with discontinuation leading to a rebound in growth of the TSC-associated lesions. Moreover, rapalog treatment has no significant effect on neurocognitive functioning or behavior in children with TSC [11]. Therefore, there is a clear need to identify novel therapeutics for treating TSC that are superior or complementary to rapalogs in terms of long-term effectiveness and efficacy toward various non-tumor CNS manifestations of TSC.

Several mouse models of TSC have provided valuable clues for neurological symptoms, but have limitations in faithfully recapitulating the human phenotypes [12]. Further, the inability to establish expandable human cell lines derived from various TSC-associated lesions, along with genetically matched control cell lines has made it difficult to define the precise pathogenic mechanisms involved in TSC. Patient-specific induced pluripotent stem cells (iPSCs) derived from somatic cells, followed by differentiation into specific cell types, are rapidly evolving to be powerful for disease modeling to study pathophysiology and to identify treatments [13–17]. More importantly, the emergence of powerful genome editing techniques has made it possible to generate isogenic pairs of disease and control human iPSCs that differ only with respect to disease-causing gene mutations [18–20]. Recent studies that employed either human embryonic stem cell lines with heterozygous or homozygous loss of *TSC2* or TSC patient iPSCs examined post-mitotic neurons and focused on later developmental processes such as dendrite outgrowth and synapse formation [21–26]. However, modeling of neurodevelopmental disorders with patient-derived iPSCs from ASD patients suggests that ASD risk genes can affect early phenotypes of neurogenesis such as the proliferation of neural progenitor cells (NPCs). Recent studies reveal that earlier developmental events, such as NPC proliferation, process outgrowth, and migration that occur prior to neuronal differentiation are also critical in disease pathogenesis of ASD and schizophrenia [27–31].

In this study, we have generated isogenic sets of iPSCs from a TSC patient harboring a germline *TSC1* mutation and have focused our efforts to examine the changes caused by either heterozygous or homozygous loss of TSC1 in NPCs. Both *TSC1*-Het and Null NPCs show enlarged cell size and mTORC1 activation when compared with the CRISPR-corrected WT, which are reversed by rapamycin. Further, we show basal activation of MEK-ERK signaling in *TSC1*-Het and Null NPCs, with further activation of ERK upon rapamycin treatment. Rapamycin also activates MNK-eIF4E signaling pathway, a regulator of 5’ cap-dependent translation. More importantly, we have observed genotype-dependent differences in early neurodevelopmental events such as aberrant NPC proliferation and neurite outgrowth, which are not affected by rapamycin treatment. Transcriptome analyses using RNA sequencing (RNAseq) revealed differential expression of genes related to ASD, ID, and epilepsy that were also altered in a genotype-dependent manner. Taken together, our results authenticate that both heterozygous and homozygous loss of TSC1 influence phenotypes, signaling, and gene expression in NPCs compared to the genetically matched control WT cells, supporting that heterozygous loss of *TSC1/2* may indeed play a role in some of the neurological manifestations of TSC.

## Methods

### Human iPSC line derivation

TSC1 skin fibroblast samples were collected through the TSC Clinic at Massachusetts General Hospital (Boston, USA). Genomic DNA from fibroblasts was extracted, and a germline mutation in *TSC1* exon 15 was identified using standard PCR and Sanger sequencing methods as previously described [32]. The *TSC1* heterozygous fibroblasts were cultured in high glucose DMEM (Gibco) supplemented with 15% of fetal bovine serum (Sigma) and streptomycin/penicillin (Cellgro). Cells were reprogrammed using a synthetic modified mRNA-based method to obtain iPSC lines as described [33, 34]. Briefly, cells were transfected by nucleofection (Amaxa Nucleofector I) with in vitro transcribed mRNAs encoding *OCT4*, *SOX2*, *KLF4*, *cMYC*, and *LIN28* (Stemgent). After picking clones, iPSC colonies were cultured in feeder-free culture conditions on Geltrex (ThermoFisher)-coated plates in Essential 8 medium (Gibco). Colonies were passaged every 4–6 days and the media was changed daily. We also utilized an unrelated wildtype control iPSC line 8330 that was originally generated from fibroblasts (GM08330) obtained from the Coriell Institute for Medical Research) as previously reported [35]. Karyotyping was performed by WiCell Cytogenetics Lab (Madison, WI). Sanger sequencing was performed by Eton Bioscience (San Diego, CA).

### CRISPR/Cas9 method for generating isogenic iPSC lines

To generate isogenic iPSC lines (Corrected-WT and Null), CRISPR/Cas9 genome editing was performed employing *TSC1* heterozygous patient-derived iPSCs. For the TSC1-Null lines, a single guide RNA (sgRNA) sequence was designed to target *TSC1* exon 7 (sgRNA seq: GAGATAGACTTCCGCCACG). For CRISPR-mediated corrections, a sgRNA was designed to specifically target the mutant *TSC1* exon 15 allele (sgRNA seq: GGGAGACTGTCTCAGTAAA) to correct the germline microdeletion mutation. sgRNAs were cloned into the pSpCas9(BB)-2A-Puro (PX459) vector and plasmids were prepared using an endo-free midi prep (Qiagen). To achieve a gene knock-in to correct the mutation on exon 15, we used a 99 base pair wildtype donor sequence designed for homologous recombination of the allele bearing the mutation. iPSCs were pre-incubated with ROCK-inhibitor at 10 μM for 2 h prior to nucleofection with 1 μg of vector using the human stem cell nucleofector kit I (Lonza) with the Amaxa Nucleofector I (program B-16). Cells were then plated and selected on the following day with 0.5 μg/ml of puromycin for 2 days. Selected cells were grown for 10 days allowing colonies to form. Colonies were manually isolated in Geltrex-coated 48 well plates and expanded. After 3–4 days, genomic DNA was extracted from a portion of each colony followed by Sanger sequencing using targeted primers (see Additional file 3: Table S2). Clones showing successful gene editing were then further expanded and used for subsequent experiments.

### Differentiation of iPSCs into NPCs

TSC1-iPSC lines were differentiated using the directed monolayer differentiation protocol [36]. Briefly, iPSCs expressing the pluripotency marker TRA-1-60 were sorted and enriched using the MACS Microbead cell sorting technology (Miltenyi Biotec) and plated in feeder-free conditions at a density of 2 − 2.5 × 10^4^ cells per cm^2^. Cells were cultured in neural induction media (neurobasal media supplemented with 1× neural induction supplements (Gibco)) for 7–9 days, after which they expressed polysialylated-neural cell adhesion molecule (PSA-NCAM). The PSA-NCAM-positive (+) cells were first isolated by MACS sorting, and then double sorted to enrich for NPCs representing CD271^-^/CD133^+^ cells. NPCs were then cultured in neural expansion media (50% neurobasal media and 50% advanced DMEM/F12 (Gibco) supplemented with 1× neural induction supplements (Gibco)) up to 15 passages and periodically assessed for expression of NPC markers, NESTIN, and SOX2.

### Neurite outgrowth assay

NPCs were seeded on Poly-D-Lysine (0.1 mg/ml, Sigma) and Fibronectin (5 μg/ml, Corning) coated wells at 6250 cells per cm^2^ in growth factor depleted Neural Expansion Medium (30% NEM) containing 49.7% neurobasal media, 49.7% advanced DMEM (Gibco), 1× penicillin/streptomycin and 0.3× neural induction supplements. Cells were grown for 48 h and fixed with 4% paraformaldehyde (PFA) for 20 min prior to immunostaining. Four independent field images with approximately 50 cells per field were analyzed. Processes that were at least two times the length of the cell body were considered as neurites. The average neurite number per cell and the average longest neurite length per cell were analyzed using HCA-Vision software V2.2.0 (CSIRO).

### Immunocytochemistry

Cells were fixed with 4% paraformaldehyde for 20 minutes at room temperature. Non-specific labeling was blocked using 4% Normal Goat Serum in PBS with 0.1% of Triton-X-100 for 45 min at room temperature. Primary antibodies were diluted into 2% NGS-PBS-Triton-X 0.1% and incubated overnight at 4°C (see Additional file 2: Table S1). Secondary antibodies were diluted into 2% NGS-PBS-Triton-X 0.1% and incubated for 2 h in the dark at room temperature (see Additional file 2: Table S1). DAPI was used to stain nuclei (Invitrogen #D3571) at 5 μg/ml. Coverslips were mounted in ProLong Gold Antifade Mountant (Invitrogen #P36930) and images were captured using a Nikon Eclipse TE2000-U microscope and the NIS-Element BR 3.2 imaging software.

### Immunoblot analyses

Cells were lysed in RIPA buffer as previously described [37, 38]. Protein lysates were resolved on 4–20% Criterion^TM^ TGX^TM^ gel (BioRad), transferred to nitrocellulose (Biorad) and then incubated with primary antibodies (see Additional file 2: Table S1). All immunoblotting data shown is a representative of 3 biological replicates.

### Quantitative RT-PCR

Total RNA from iPSCs was isolated by lysis in TRIzol reagent (Ambion/Life Technologies; Grand Island, NY) according to the manufacturer’s instructions. Following lysis, RNA was rinsed in chloroform, and the aqueous layer was applied to Qiagen RNeasy kit (Qiagen) columns followed by purification according to manufacturer’s instructions. For cDNA synthesis, the Superscript VILO cDNA synthesis kit (Life Technologies) was used according to the manufacturer’s instructions, and quantitative RT-PCR (q-RT-PCR) was carried out using TaqMan according to manufacturer’s instructions.

### RNA-seq library preparation and sequencing

Total RNA was isolated from NPC lines using TRIzol reagent. Briefly, pelleted cells were resuspended in TRIzol reagent and then extracted with chloroform, followed by isopropanol precipitation of RNA from the aqueous phase and three 70% ethanol washes. RNA pellets were solubilized in 30-50 μl of RNase-free water (Ambion, AM9937). RNA quality was assessed using the Agilent Bioanalyzer TapeStation 2200 (Agilent Technologies, Santa Clara CA). In total, 12 RNA-seq libraries were prepared in triplicate for each of the four NPC samples harboring WT, Het TSC1, and two clones of null TSC1 using the Illumina TruSeq Stranded mRNA Sample Prep Kit. Each library in this study included 1 μl of a 1:10 dilution of ERCC RNA Control Spike-Ins (Ambion) that were added from one of two mixes, each containing the same 92 synthetic RNA standards of known concentration and sequence. These synthetic RNAs cover a 10^6^ range of concentration, as well as varying in length and GC content to allow for validation of dose-response and the fidelity of the procedure in downstream analyses [39]. Libraries were multiplexed, pooled, and sequenced on multiple lanes of an Illumina HiSeq2500, generating median 74.5 M paired-end reads per library of 100 bp.

### RNA-seq data processing and analysis

Quality checking of sequence reads was assessed using fastQC (v.0.10.1) (http://www.bioinformatics.babraham.ac.uk/projects/fastqc). Sequence reads were aligned to the human reference genome (GRCh37, Ensembl build v. 75) using STAR (version 2.5.2a) with parameters ‘–outSAMunmapped Within –outFilterMultimapNmax 1–outFilterMismatchNoverLmax 0.1–alignIntronMin 21–alignIntronMax 0–alignEndsType Local–quantMode GeneCounts–twopassMode Basic’ [40]. STAR aligner also generated gene-level counts for all libraries relying on the human gene annotation provided for Ensembl GRCh37, build 75. Based on quality checking of alignments assessed by custom scripts utilizing PicardTools (https://broadinstitute.github.io/picard/), RNASeQC [41], RSeQC [42], and SamTools [43], no outlier sample was identified. Differentially expressed genes (DEGs) in three pair-wise comparisons including Het vs. WT, Null_A vs WT, and Null_B vs WT were identified by edgeR’s quasi-likelihood F test (v. 3.18.1) [44], testing differential expression between selected TSC1-mutant samples, Het or Null, and WT samples, which was run at the R platform (v. 3.4). In differential expression analysis, genes that passed the expression detection threshold, which was determined to be > 5 based on ERCC analysis as described in [45], in at least half of six analyzed samples in a given comparison were analyzed. Further comparison analysis revealed that 107 DEGs with Bonferroni adjusted *p* values < 0.05 overlapped among three pair-wise comparisons, of which 29 DEGs (9 upregulated, 20 downregulated) showed dosage effect in which significantly up- or downregulated DEGs with more than 2-fold changes in Het vs. WT comparison showed at least 1.5-fold up or downregulation in each Null vs. WT comparisons compared to their fold change in Het vs. WT. Gene ontology (GO) enrichment analysis was performed separately for 9 up-regulated and 20 downregulated DEGs represented by Ensembl gene IDs, using R/Bioconductor topGO package (v. 2.28) in R with “weight01” algorithm and “fisher” statistics and nodeSize = 10 relying on genome-wide annotations for Human in R/Bioconductor package org.Hs.eg.db (v. 3.4.1). In the GO enrichment analysis, union of all the analyzed genes in each of three comparisons, containing 22,235 genes were used as a background gene set.

## Results

### Generation and characterization of an isogenic set of *TSC1*-iPSC lines

**Fig. 1:**
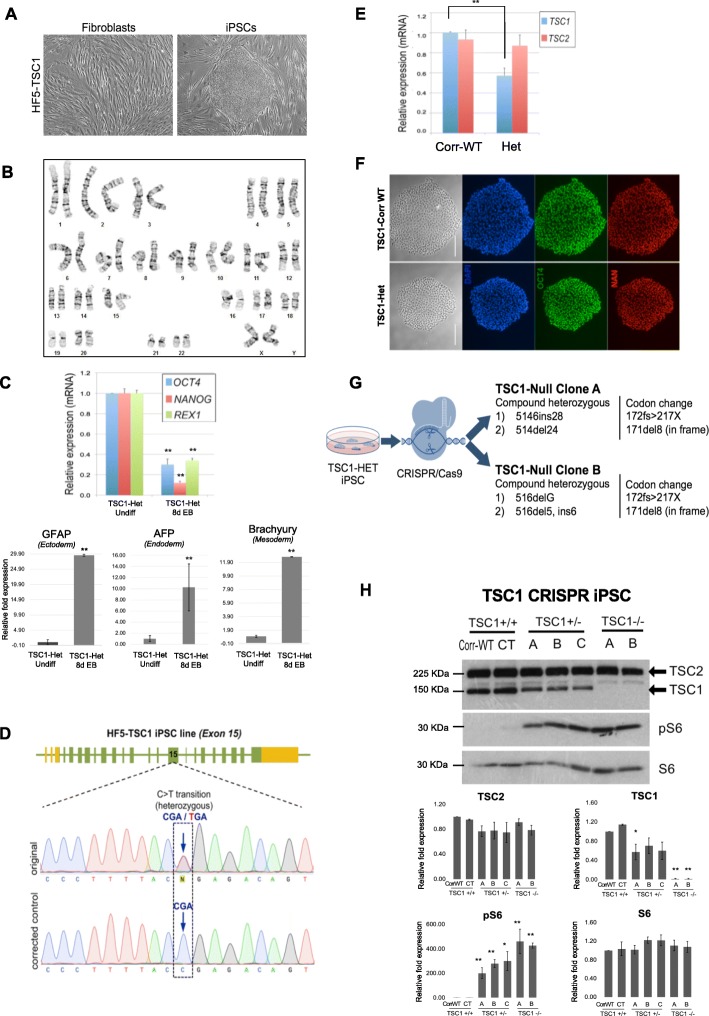
Generation and characterization of isogenic TSC1-iPSCs. **a** Bright field images of a TSC1-iPSC colony (right) generated from skin fibroblasts of a TSC1 patient (left). **b** Representative normal karyotype of heterozygous TSC1-iPSCs. A total of 20 cells were counted to confirm normal diploidy of 46 and a total of 8 cells were analyzed in which the chromosomes were compared band by band to their homologues and a total of 4 were karyotyped. **c** Upon differentiation, real-time PCR in embryoid bodies (EB) from TSC1-iPS cells show decreased pluripotency markers (*OCT4*, *NANOG*, and *REX1*) at 8 days post-differentiation (8dEB) versus undifferentiated iPSCs and increased expression of the 3 germ layers markers (*GFAP* for ectoderm, *AFP* for endoderm, and *Brachyury* for mesoderm). Error bars represent standard deviation on 3 biological replicates. Data were normalized to the undifferentiated control. Mean values are shown, **p < 0.001 calculated with Student’s *t* test **d** CRISPR/Cas9-mediated correction of TSC mutation. Sequencing of original and corrected control iPSC lines derived from a TSC1 patient. **e** Quantitative RT-PCR shows increased *TSC1* (left) expression in CRISPR-corrected iPSCs (Corr-WT) compared to heterozygous (Het) original iPSCs. Data were normalized to the Corr-WT. Mean values are shown, error bars represent standard deviation of 3 biological replicates, **p < 0.001 calculated with Student’s *t* test. No change in expression of *TSC2* was noted. **f** Isogenic iPSCs heterozygous (Het) and corrected (Corr-WT), immunostained for pluripotency markers OCT4 (green) and NANOG (NAN, red). Bright field and nuclear DAPI (blue) are also shown. Scale bar = 200 μm. **g** Schematic representation of the two TSC1-Null clones of iPSCs generated using CRISPR/Cas9. **h** Immunoblot of TSC1 iPSC lines compared to an unrelated TSC iPSCs control (CT). Expression of TSC1 is reduced in TSC1-Het clones and completely lost after introducing a second somatic mutation, and mTORC1 is activated in both the Het and Null clones as shown by elevated expression of pS6. **a**–**c** Independent iPSC clones of TSC1-Het (TSC1+/−) or TSC1-Null (TSC1−/−). Expression of TSC2, TSC1, phosphorylated S6 (pS6), and total amount of S6 (S6) were quantified from at least 3 independent experiments. Data were normalized to the Corr-WT. Mean values ± S.D. of three separate experiments are shown, *p < 0.01, **p < 0.001 calculated with Student’s *t* test

We established a TSC patient-derived iPSC line by reprogramming skin fibroblasts bearing a truncating nonsense mutation in exon 15 of *TSC1* (1746C>T, Arg509X). We used a non-viral, synthetic modified mRNA-based method eliminating the risk of genomic integration and/or mutagenesis inherent to DNA-based approaches [46]. iPSCs showed typical colony formation in three independent clones (Fig. 1a), a normal karyotype (Fig. 1b), an expected decrease in the expression of pluripotency markers (*OCT4*, *NANOG*, and *REX1*) upon differentiation to embryoid bodies (EB). Further EB assays performed as recently described to assess the differentiation potential of iPSCs [47] revealed an increase in the expression of the three germ layer markers representing ectoderm (*GFAP)*, endoderm (*AFP*), and mesoderm (*Brachyury*) (Fig. 1c, 3 biological replicates).

We then used the CRISPR/Cas9 technique to correct the mutation in *TSC1*-Het iPSCs by using a mutant specific sgRNA and a single-stranded oligo donor (ssODN) (Fig. 1d and Additional file 3: Table S2). The resulting wildtype iPSC line with the same genetic background as the original patient-derived TSC1-Het iPSCs is referred to as corrected wildtype (Corr-WT) and is used as a control. Real-time PCR analysis of Corr-WT TSC1-iPSCs showed increased expression of *TSC1* compared to the original *TSC1*-Het line (Fig. 1e). We tested *TSC2* mRNA expression since loss of *TSC1* could affect the expression of *TSC2* and observed no significant difference in expression of *TSC2* mRNA between TSC1-Het and Corr-WT (Fig. 1e), all performed in three biological replicates. iPSC colonies were assessed for pluripotency by immunostaining and showed normal expression of OCT4 and NANOG (Fig. 1f). We also confirmed that the differentiation capacities of the Corr-WT iPSCs were intact by observing a decrease in pluripotent marker expression during embryoid body formation, and cytogenetic analysis revealed normal karyotyping (data not shown). To represent a second somatic mutation in *TSC1*, employing the CRISPR/Cas9 method again, we introduced mutations in the TSC1-Het iPSC line by targeting exon 7 using sgRNA cloned in pSpCas9(BB)-2A-Puro (PX459) vector (Additional file 3: Table S2). Two independent clones A and B with compound heterozygous mutations in *TSC1* exon 7 (Fig. 1g) and showing normal OCT4 expression (Additional file 4: Figure S1) were chosen for further investigation. Immunoblotting for TSC1/hamartin protein in Corr-WT (*TSC1*+/+) showed expression level similar to another unrelated control iPSC line (CT), while the expression was reduced in three independent TSC1-Het iPSC clones (A, B, and C) and completely lost in TSC1-Null mutant iPSC lines. TSC2 expression was not changed significantly in either TSC1-Het or TSC1-Null iPSC lines when compared with the Corr-WT. As predicted, mTORC1 signaling was activated as shown by elevated expression of readout phosphorylated S6 (pS6) in both TSC1-Het and Null iPSC lines (Fig. 1h). These results were obtained on at least three independent experiments and confirmed the successful generation of an isogenic set of iPSCs representing TSC1 Corr-WT, Het, and Null, and we chose one clone for each genotype for all subsequent experiments.

### Differentiation of isogenic TSC1-iPSC lines into NPCs

**Fig. 2:**
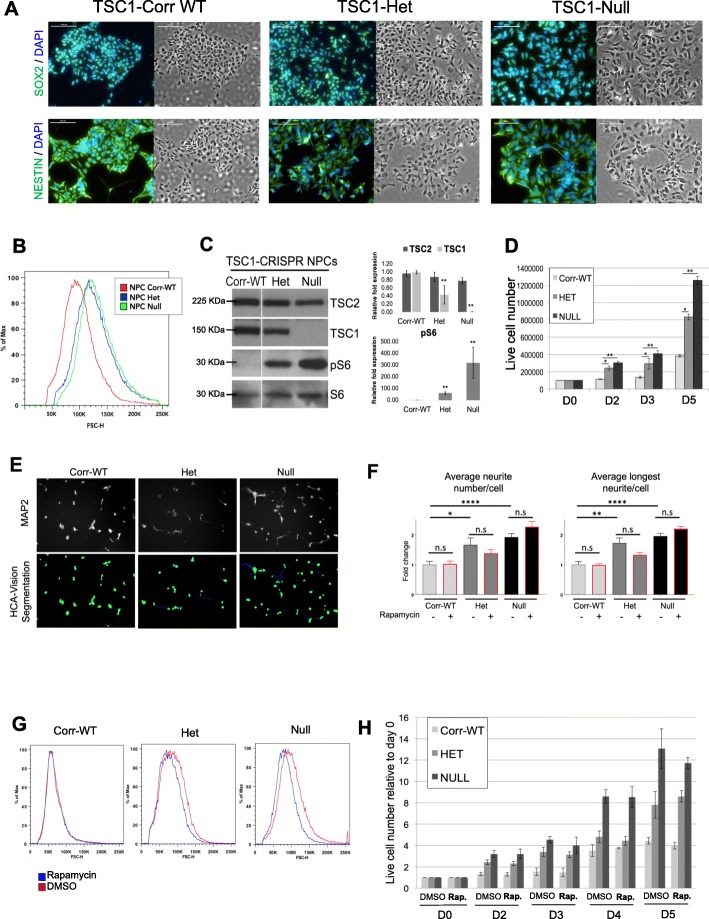
Characterization of TSC1 iPSC-derived NPCs. **a** All TSC1 NPC lines (Het, Null, Corr-WT) express expected neural progenitor markers SOX2 (upper panel) and NESTIN (panel below). DAPI in blue, SOX2 in green, and NESTIN in green. Scale bar = 100 μm. Immunostaining was performed at least 3 times. **b** TSC1-Het and Null NPCs display increased cell size compared to TSC1-Corr-WT as shown in bright field images (**a**) and by forward scatter FACS analysis; *n* = 3. **c** As expected, TSC1-Null and -Het NPCs show dose-dependent increased mTORC1 signaling (pS6 readout) compared to Corr-WT. Protein expression was quantified and normalized to the Corr-WT NPCs, *n* = 6, mean values ± s.e.m. are shown, *p < 0.01, **p < 0.001 calculated with Student’s *t* test. **d** Proliferation rate of NPC lines was quantified at day 0 (D0, equal cell seeding), and live cell numbers were assessed at D2, D3, and D5. Mutant TSC1 NPCs (Het and Null) revealed genotype-dependent increased proliferation compared to Corr-WT. Data was normalized to Corr-WT at D0, mean values ± S.D. of three separate experiments are shown,*p < 0.01, **p < 0.001 calculated with Student’s *t* test. **e**, **f** MAP2 immunostaining showed genotype-dependent increased neurite outgrowth (number and length) in TSC1 mutant NPCs, which were quantified using a custom image analysis pipeline and HCA Vision imaging software creating neurite segmentation (representative panel shown for the DMSO-treated NPCs). Analysis on *n* = 6 field images per treatment with approximately 50 cells per field. Data normalized to DMSO treated Corr-WT NPCs. Mean values + s.e.m. are shown. *p < 0.05, **p < 0.001, ****p < 0.0001, n.s. = not significant, calculated with Welch’s *t* test (GraphPad Prism 7.05). **g** TSC1 NPCs (Corr-WT, Het, and Null-clone B) were treated with 100 nM of rapamycin for 24 h or with DMSO and analyzed by flow cytometry using the forward scatter height (FSC-H) gating. For each cell line the blue curve represents the rapamycin-treated cells and the red curve represents the DMSO control cells. A shift in the curves shows a cell size difference. *N* = 3. **h** Proliferation rate of NPC lines after treatment with a vehicle control (DMSO) or rapamycin (100nM) was quantified at day 0 (D0, equal cell seeding), and live cell numbers were assessed at D2, D3, D4, and D5. No significant differences were observed between DMSO or rapamycin-treated NPCs in all cell types at all time point; *n* = 3. Mean values ± S.D. of three separate experiments are shown, data was normalized to the Corr-WT treated with DMSO at D0

To generate a pure population of stable and expandable NPCs from iPSCs, we adapted a recently published protocol of directed monolayer differentiation using microbead sorting based on the expression of neural cell surface protein markers [36]. Here, we first pre-selected iPSCs based on the expression of an immature embryonic stem cell surface pluripotency marker (TRA-1-60) to maximize efficiency. We next selected PSA-NCAM-positive (PSA-NCAM^+^) cells to enrich for developing neuronal lineage cell populations and then sorted for CD133^+^/CD271^−^ cells to isolate NPCs and eliminate the neural crest cell population. Following microbead purification, the resulting isogenic set of iPSC-derived TSC1-NPCs was confirmed by immunofluorescent staining for neural markers SOX2 and NESTIN at least 3 times for each genotype. (Fig. 2a).

### TSC1 iPSC-derived NPCs show genotype-dependent phenotypes

We examined morphological differences between TSC1-CorrWT, TSC1-Het, and TSC1-Null NPCs, and found that TSC1-Het and Null NPCs were larger in size than the Corr-WT on 3 biological replicates and a representative image is shown in Fig. 2b. This is consistent with the reduction or loss of TSC1 in the Het and Null respectively, inducing a strong activation of mTORC1 as seen on at least six independent replicates by higher phospho-ribosomal protein S6 (pS6) expression levels in a dose-dependent manner (Fig. 2c). TSC2 expression is included as a control (Fig. 2c). We also observed that both the TSC1-Het and Null NPCs proliferate faster by day 5 when compared with the matched Corr-WT as determined by viable cell counts using trypan blue exclusion and automated cell counting on three independent experiments (Fig. 2d).

Previous studies of neurodevelopmental disorders including Rett Syndrome, Fragile-X Syndrome, TSC, ASD, and schizophrenia have used post-mitotic neurons derived from human iPSCs or mouse models to study morphological aspects such as dendrite outgrowth or synapse formation, or functional characteristics using neuronal electrophysiology, which represents late-stage neurodevelopmental events. However, NPC proliferation and initial process extension phenotypes, such as neurite number and length represent earlier events in neurogenesis and are strongly implicated in ASD [29, 30]. Therefore to explore the potential early neurodevelopmental deficits, we examined the isogenic NPC set using phalloidin and MAP2 immunostaining to quantify neurite length, number and branching points and to compare the genotype-specific differences. Interestingly, TSC1-Het and Null NPCs revealed a significant increase in average neurite number as well as average neurite length per cell (*n* = 6 field images for each genotype with approximately 50 cells per field). (Fig. 2e, f). Rapamycin treatment did not have an effect on neurite number or length (*n* = 3) (Fig. 2f). Rapamycin treatment (100 nM) for 24 h reduced cell size in TSC1-Het and Null (Fig. 2g) but had no effect on proliferation as shown by viable cell counts (*n* = 3) (Fig. 2h) and confirmed by flow cytometric analysis of cell cycle using quantification of propidium iodide DNA staining or a Cell-Titer-Glo viability assay that assess cellular ATP levels (Additional file 1: Supplementary Materials and Methods; Additional file 4: Figure S2).

These data suggest that early neurodevelopmental phenotypes, such as proliferation and neurite outgrowth that occur prior to neuronal differentiation are altered in TSC1-Het and Null NPCs when compared with the matched WT and that mTORC1 inhibition though rapamycin treatment does not affect these phenotypes.

### TSC1-mutant NPCs reveal activation of MEK-ERK signaling

**Fig. 3:**
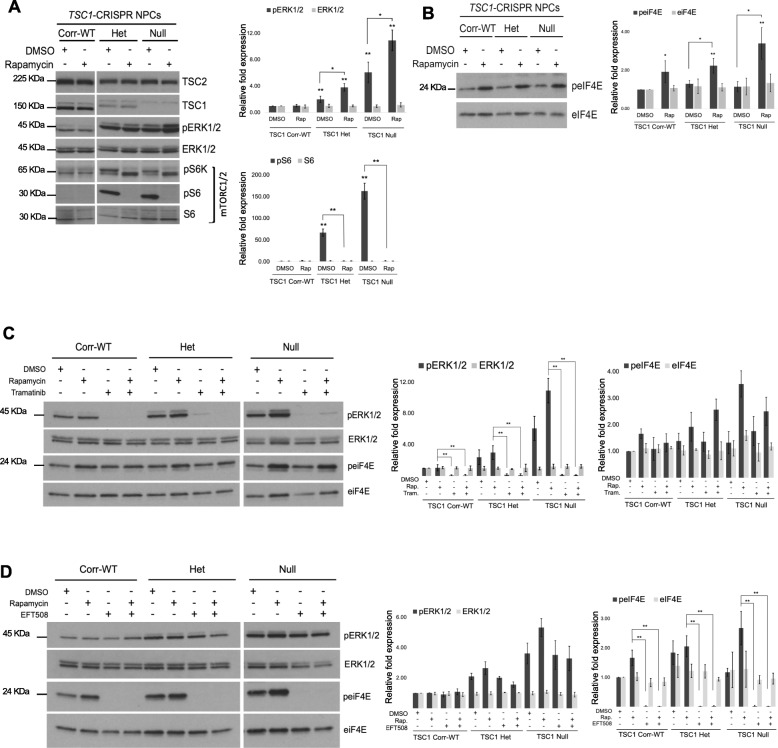
Activation of MEK/ERK and MNK-eIF4E pathways in TSC1 mutant NPCs. **a** In addition to mTORC1/2 activation, immunoblotting also showed Increased phosphorylation of ERK1/2 (pERK1/2) in TSC1-Het and Null NPCs. As predicted, rapamycin (100 nM, 24 h) inhibited mTORC1 signaling (pS6K and pS6 readouts). Rapamycin increased pERK1/2 in TSC1-Null NPCs; *n* = 5. **b** Treatment with rapamycin (100 nM, 24 h) leads to increased phosphorylation of translational subunit eIF4E (p-eIF4E) in TSC1-NPCs; *n* = 6. **c** Treatment with a MEK inhibitor tramatinib (250 nM, 24 h) alone or combined with rapamycin (100 nM, 24 h) inhibits phosphorylation of ERK1/2 (pERK1/2) but has no effect on the translational subunit eIF4E; *n* = 3. **d** Increased p-eIF4E in TSC1-Het and Null NPCs is blocked using MNK inhibitor eFT508 (50 nM, 24 h), alone or combined with rapamycin (100 nM, 24 h); *n* = 3. Mean values ± S.D. of three separate experiments are shown, *p < 0.01, **p < 0.001 calculated with Student’s *t* test

We have previously reported MEK-ERK signaling to be aberrantly elevated in TSC patient subependymal giant cell astrocytoma cells (SEGAs) compared to normal brain [48]. Therefore, we investigated whether TSC1-mutant NPCs had similar dysregulation of this pathway. Interestingly, we observed at least in five independent experiments an increase in pERK1/2 in TSC1-Het and TSC1-Null NPCs compared to Corr-WT (Fig. 3a), which is consistent with our previous report and differs from *Tsc1/2*-Null MEFs where ERK signaling is downregulated due to feedback inhibition by Grb10 [49]. Our results suggest that in addition to mTORC1 signaling, MEK-ERK signaling is upregulated in TSC1-mutant human neuronal cell types. Rapamycin treatment, as expected, blocked mTORC1 activation as detected by reduced phospho-ribosomal protein S6 kinase (pS6K) and pS6 levels, (Fig. 3a). Rapamycin however significantly increased pERK1/2 in TSC1-Het and Null NPCs (Fig. 3a).

Recent studies demonstrated that a single, conserved Ser residue (S209) in the Eukaryotic Translation Initiation Factor 4E (eIF4E), phosphorylated by MAP kinase-interacting kinase (MNK) [50] not only plays a role in cancer biology, but also in neurobiology by regulating 5’ cap-dependent translation of specific mRNAs in neuronal cells [51]. Rapamycin is known to increase eIF4E phosphorylation at S209 in many cancer cell types [50, 52, 53]. To our knowledge, the phospho-status of eIF4E has not been assessed with TSC1/2-deficiency. In our TSC1 NPCs, we found no significant basal activation; however, upon treatment with rapamycin, we observed upregulation of p-eIF4E S209 (*n* = 6) (Fig. 3b). These results suggest that mTORC1 inhibition in TSC1-mutant NPCs leads to increases in both MEK-ERK and MNK-eIF4E signaling pathways, both of which are known to regulate protein translation [6, 51, 54].

To understand the mechanism of rapamycin-induced activation of ERK1/2 and eIF4E in TSC1 NPCs, we examined the effects of a MEK inhibitor trametinib, and a MNK inhibitor eFT508 (tomivosertib), which is currently in clinical development, either alone or in combination with rapamycin. Trametinib treatment inhibited phosphorylation of ERK1/2 as expected, but had no effect on p-eIF4E (*n* = 3) (Fig. 3c). Conversely, eFT508 treatment completely inhibited p-eIF4E but not pERK1/2 (*n* = 3) (Fig. 3d). These results suggest that phosphorylation of eIF4E by MNK is not regulated by MEK-dependent ERK signaling in TSC1 NPCs.

### Rapamycin induced activation of eIF4E is partly dependent on PI3K activation

**Fig. 4:**
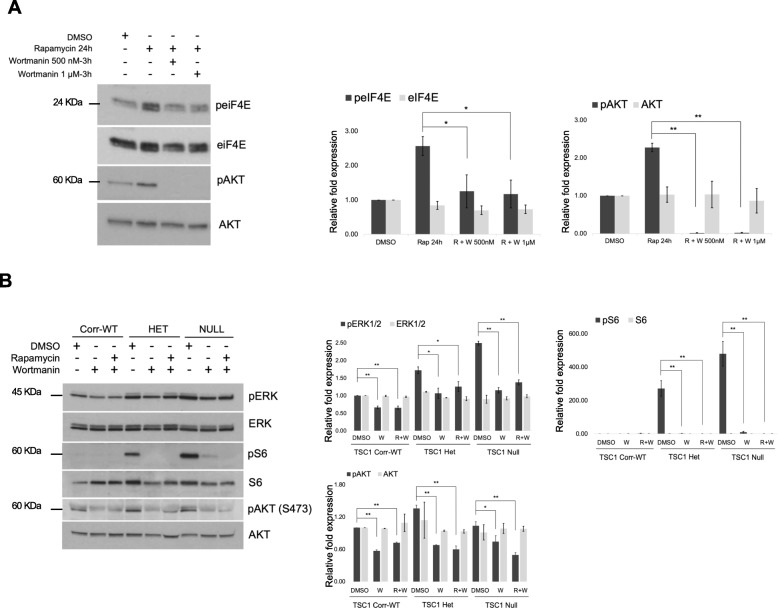
Rapamycin induced activation of eIF4E and ERK1/2 is partly dependent on PI3K activation. **a** Treatment with wortmannin (500 nM or 1 μM for 3 h) combined with rapamycin (100 nM for 24 h) in TSC1-Null NPCs decreases phosphorylation of eIF4E; *n* = 3. **b** Treatment with wortmannin (1 μM for 3 h) alone or in combination with rapamycin (100 nM for 24 h) inhibits pAKT (s473) and decreases phosphorylation of ERK1/2 in TSC1 mutant NPCs; *n* = 3. pS6 serves as a control. For each panel, protein expression was quantified and normalized to the Corr-WT NPCs treated with DMSO. Mean values ± S.D. of three separate experiments are shown, *p < 0.01, **p < 0.001 calculated with Student’s *t* test

We observed that the increase in p-eIF4E after rapamycin treatment was more pronounced in TSC1-Null NPCs that have strong activation of mTORC1 (*n* = 3) (Fig. 3c, d), suggesting that inhibition of mTORC1 may relieve the negative feedback regulation on PI3K signaling, resulting in an increase in p-eIF4E. Further, studies performed in human cancer cells have shown that MNK-dependent eIF4E phosphorylation is regulated by PI3K signaling [52, 53]. Therefore, we examined the effects of a PI3K inhibitor wortmannin and observed that increased eIF4E phosphorylation after rapamycin treatment is partly dependent on PI3K signaling (*n* = 3) (Fig. 4a). Similarly, rapamycin-induced increase in pERK1/2, particularly in TSC1-Null NPCs was partially inhibited by wortmannin supporting a PI3K-ERK signaling axis in TSC1 mutant NPCs (*n* = 3) (Fig. 4b).

### Transcriptome analyses of TSC1 isogenic NPCs

**Fig. 5:**
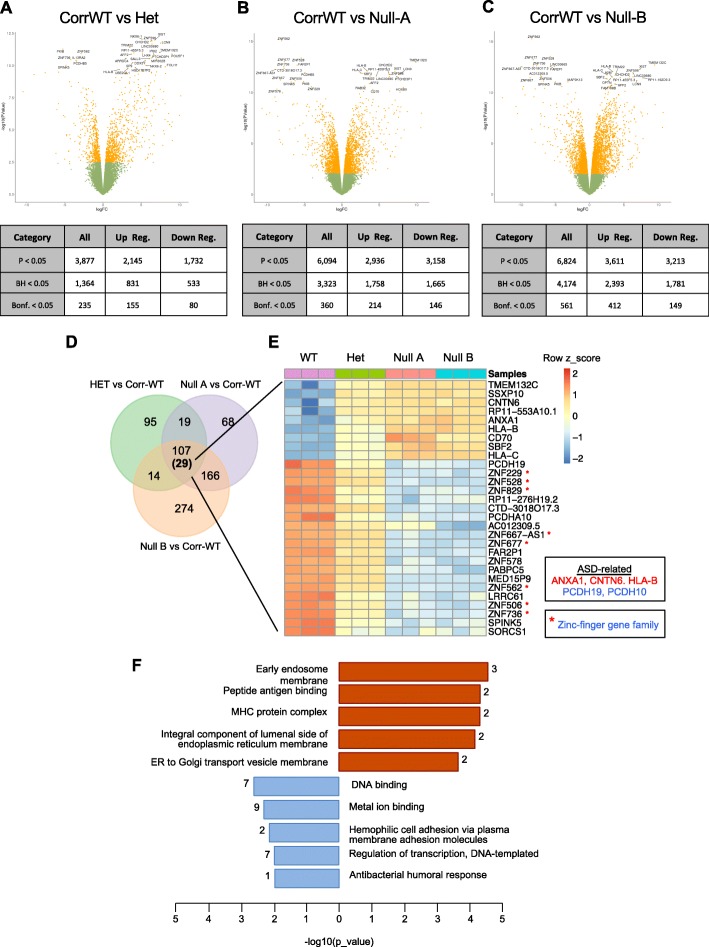
Transcriptome analysis of TSC1 NPCs reveals genotype-dependent changes in gene expression. **a–c** Differential expression analysis identified distinct and shared upregulated and downregulated genes in the isogenic TSC1 NPC set for Het or 2 independent Null clones (**a**, **b**) versus Corr-WT. **d** Of the 107 genes shared between Het and Null NPCs, 29 showed a genotype-dependent linear response (showed on the heat map: **e**). This subset included ASD-related genes (red, up; blue, down) as well as multiple members of the Zinc-finger gene family of transcription factors (*). **f** Gene ontology analysis for the genes that showed a linear response

To further characterize the NPCs at the transcriptome level, we performed RNA-seq of TSC1 NPCs representing Corr-WT, Het, and two independent Null clones (Null-A and Null-B) in triplicates. Differential expression analyses of these samples identified distinct and shared upregulated or downregulated genes between TSC1-Het versus Corr-WT, and TSC1-Null (A and B) versus Corr-WT (Fig. 5a–c). We observed a total of 107 differentially expressed genes that overlapped between Het and Null, when compared with the Corr-WT at a high stringency of analyses (Bonferroni adjusted *p* < 0.05) (Fig. 5d). It is noteworthy that of the 107 genes, 29 displayed a genotype-dependent linear response, i.e., genes upregulated or downregulated in TSC1-Het NPCs that were further increased or decreased in TSC1-Null NPCs, respectively. In particular, genes such as *ANXA1*, *CNTN6*, *HLA-B*, *PCDH19*, and *PCDH10*, have been linked to ASD, epilepsy, ID, and other neuropsychiatric disorders [55–60], were significantly upregulated or downregulated and warrant further investigation (Fig. 5e). In addition, several genes encoding members of the Zinc-finger protein (ZNF) family of sequence-specific, DNA binding transcription factors [61] were downregulated in TSC1-Het and Null NPCs raising the possibility of their role in neurodevelopment. Gene ontology analyses for TSC1-Het and Null NPCs revealed enriched categories for upregulated genes including endosome membrane, endoplasmic reticulum (ER), and ER-to-Golgi transport vesicle membrane, whereas downregulated genes revealed enrichment related to DNA binding and regulation of transcription (Fig. 5f). Taken together, these data support the notion that loss of TSC1 causes aberrant changes in the transcriptome and in changes in pathways critical for shaping the neural proteome relevant for early neurodevelopment.

## Discussion

Neurodevelopmental syndromes including ASD, Fragile X, and TSC are commonly considered disorders of synaptic homeostasis and therefore, several prior studies have focused on differentiated neurons and synaptic defects. However, emerging studies reveal early neurodevelopmental events such as NPC proliferation, neurite outgrowth, and migration that precede synaptogenesis also play a vital role in disease pathogenesis of ASD and other neuropsychiatric disorders [27–31, 35, 62]. Further, exome sequencing studies in ASD as well as network analyses of large numbers of ASD-implicated genes also indicate that defects in neural progenitor cell division could be a shared phenomenon regulated by these genes [63–66]. Recent studies in TSC that employed either human embryonic stem cell lines with heterozygous or homozygous loss of *TSC2* or TSC patient iPSC-derived neurons with heterozygous loss of *TSC2* confirmed that mTORC1 inhibition corrects synaptic defects [22, 25]. However, while the treatment of TSC patients with an mTORC1 inhibitor manages tumor growth, its effectiveness in treating TSC-associated neuropsychiatric defects has remained equivocal [11]. Clinical studies and mouse models provide strong evidence to support the hypothesis that the NPC lineage is the cell of origin for the CNS manifestations of TSC, and the neural crest cell (NCC) lineage is responsible for other aspects of TSC [67]. To further elucidate these mechanisms, here we have generated a cellular model using NPCs to study early neurodevelopmental aspects of TSC. With an isogenic set of NPCs (Corr-WT, Het, and Null) derived from TSC patient iPSCs with heterozygous loss of TSC1, we focused our characterization on neurogenesis phenotypes such as NPC proliferation and neurite outgrowth. Our results convincingly show enhanced proliferation in TSC1-Het and Null NPCs when compared with the isogenic control (Corr-WT), which is consistent with previous reports [23, 25]. However, unlike the previous studies, rapamycin treatment did not have an effect on enhanced proliferation and neurite outgrowth in TSC1-Het and Null NPCs when compared to the Corr-WT, suggesting that early neurodevelopmental phenotypes seen upon loss of *TSC1* are not solely dependent on mTORC1 activation. Thus, our isogenic TSC1 NPC cell models provide an opportunity to screen for drugs that could reverse the early neurodevelopmental phenotypes such as NPC proliferation and neurite outgrowth, which may ultimately lead to better treatment for TSC-associated epilepsy and neuropsychiatric defects.

**Fig. 6:**
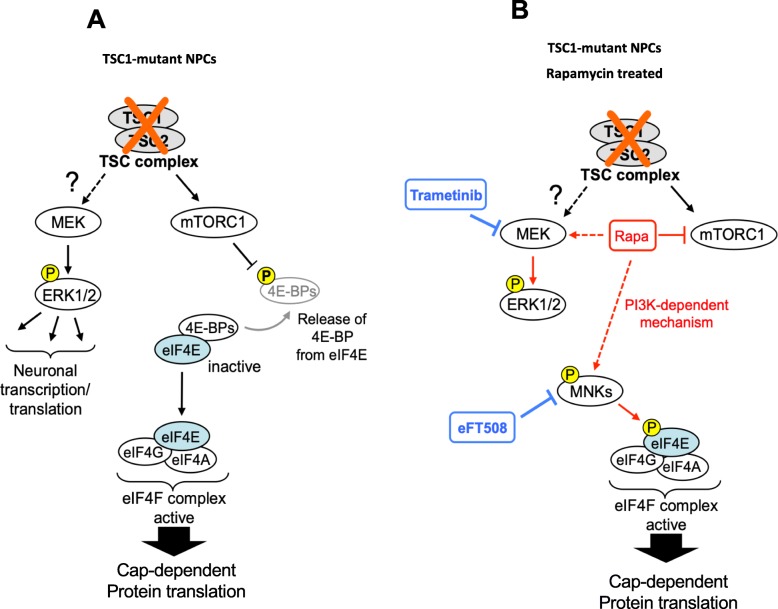
Model depicting downstream signaling pathways in untreated and rapamycin-treated TSC1-mutant NPCs. **a** In untreated TSC1-mutant NPCs, loss of the TSC protein complex results in activated MEK-ERK and mTORC1 signaling. Activated mTORC1 leads to phosphorylation and release of the inhibitory 4EBP1 from the eIF4E subunit enabling the formation of an active eIF4F complex. **b** Upon rapamycin (Rapa) treatment, while mTORC1-dependent eIF4E activation is inhibited, an alternate mechanism of MNK-mediated eIF4F phosphorylation/activation is enhanced, which is blocked by co-treatment with the MNK inhibitor eFT508

MNK-dependent phosphorylation of eIF4E S209 plays a role in neurobiology by regulating the translation of specific mRNAs in neuronal cells [51]. It is well established that the mTORC1 signaling pathway is critically involved in protein translation through regulation of initiation. mTORC1 activation results in hyperphosphorylated 4E-BP, which in turn releases the bound eIF4E and facilitates the assembly of eukaryotic initiation factor (eIF) 4F, a heterotrimeric complex composed of eIF4E, a cap-binding protein; eIF4A, an RNA helicase; and eIF4G, a large scaffolding protein that recruits 40S ribosomes to mRNA templates (Fig. 6a). Our results suggest that in TSC1 mutant NPCs, while rapamycin inhibits translation regulated by mTORC1 through phospho-4E-BP, it possibly enhances translation mediated by MEK-ERK and MNK-eIF4E pathways, which couples synaptic activity to the translational machinery and plays an essential role in neuronal translation. Our results also reveal that rapamycin-induced activation of MNK-eIF4E could be mediated by PI3K (Fig. 6b), consistent with prior studies in other cancer cells [52, 53]. Interestingly, the MNK-eIF4E axis controls the translation of mRNAs and synaptic plasticity through regulation of the translational repressor, Cytoplasmic Fragile X Protein-Interacting Protein 1 (CYFIPI) [51, 68, 69]. Taken together, we believe that neuronal translation mediated by MEK-ERK and MNK-eIF4E could play a role in CNS aspects of TSC. Moreover, treatment with rapalogs, the current standard of care for TSC patients, may lead to adaptive changes in neuronal cells via signaling feedback mechanisms, and future translatome (referring to mRNAs being actively translated) studies performed before and after mTOR and MNK inhibitor treatment in TSC patient-derived NPCs could be valuable in providing in-depth information regarding distinct translational changes.

It is noteworthy that our transcriptome studies on the isogenic sets of TSC1 NPCs revealed genotype-dependent alterations in the expression of 29 genes suggesting they may play an essential role in TSC pathogenesis. Genes implicated in ASD such as Annexin 1 (*ANXA1*) (50), *HLA-B* [55, 70] and *CNTN6* with deletion or duplication in a spectrum of neurodevelopmental disorders and ID [71, 72] were upregulated in TSC1-Het and Null NPCS when compared with the WT. The protocadherin genes, *PCDH10* and *PCDH19* that are linked to ASD and epilepsy [58, 73, 74] were downregulated along with several members of Zinc-finger gene family of transcription factors. Follow-up studies are necessary to define the roles of these genes in neurodevelopmental phenotypes that we observe in NPCs and to understand whether expression changes correlate at the protein level and could be dependent on mTORC1 activation.

## Limitations

A limitation of this study is not understanding the role of genes linked to ASD, epilepsy, and ID, which were identified as significantly upregulated or downregulated in NPCs with heterozygous and homozygous loss of *TSC1*. The undertaking of such studies is beyond the scope of this work and follow up studies are necessary.

## Conclusions

Our results clearly establish that both heterozygous and homozygous loss of *TSC1* influence early neurodevelopmental phenotypes, signaling, and gene expression in NPCs compared to the genetically matched WT cells. Our approach of using TSC patient’s iPSC-derived NPCs will provide a useful platform for large-scale omics and drug screening studies that may identify drugs that could be superior to rapamycin or effective in combination with rapamycin to ultimately treat TSC-associated neuropsychiatric symptoms.

## Supplementary information

**Additional file 1.** Additional Material and Methods.

**Additional file 2: Table S1.** List of antibodies used.

**Additional file 3: Table S2.** List of sgRNA or PCR primers used.

**Additional file 4: Figure S1.** Both TSC1-Null iPSC clones (A and B) are pluripotent. Immunostaining of TSC1-Null iPSC clones after introducing a second somatic mutation in the *TSC1* gene. DAPI in blue, OCT4 in red. Scale bar=500μm. **Figure S2.** Rapamycin reversed TSC1 NPC cell size but not proliferation. A. Bright field images of TSC1-Het and Null NPCs showing a decreased cell size after 24h treatment with 500 nM of rapamycin. Scale bar=100μm; n=3. B. TSC1 NPCs (Corr-WT, Het and Null-clone B) were treated with increasing concentrations of rapamycin in a 6 point, 5-fold serial dilution series (0 - 10 μM) for 72h. Cell viabilities were assessed using CellTiter-Glo assays and plotted as % viability (relative to DMSO). Dose response curve data is presented as +SEM (3 replicates/dose). C. TSC1-Null NPCs were treated with DMSO (left pannel) or 100 nM of rapamycin for 24h (right pannel) and stained with propidium iodide for cell cycle analysis. Proliferating cells are represented in the S phase of the cell cycle (red).

## Data Availability

The datasets used and/or analyzed during the current study are available from the corresponding author on reasonable request.

## Abbreviations

ASD: Autism spectrum disorder
CNS: Central nervous system
Corr-WT: Corrected wildtype
CRISPR/Cas9: Clustered regularly interspaced short palindromic repeats / CRISPR associated protein 9
CYFIPI: Cytoplasmic fragile X protein-interacting protein 1
eIF4E: Eukaryotic translation initiation factor 4E
ER: Endoplasmic reticulum
ERK: Extracellular signal-regulated kinase
Het: Heterozygous
ID: Intellectual disability
iPSC: Induced pluripotent stem cells
MNK: MAP kinase-interacting kinase
mTORC1: Mammalian/mechanistic target of rapamycin complex 1
NCC: Neural crest cells
NPC: Neural progenitor cells
PI3K: Phosphoinositide 3-kinase
pS6: Phospho-ribosomal protein S6
pS6K: Phospho-ribosomal protein S6 kinase
RNA-seq: RNA sequencing
SEGAs: Subependymal giant cell astrocytoma
ssODN: Single-stranded oligo donor
TSC: Tuberous sclerosis complex
ZNF: Zinc-finger protein
