# Supplementary material for: TSC patient-derived isogenic neural progenitor cells reveal altered early neurodevelopmental phenotypes and rapamycin-induced MNK-eIF4E signaling
Source: Mol Autism. 2020 Jan 6;11:2. doi: 10.1186/s13229-019-0311-3 (PMC6945400; doi:10.1186/s13229-019-0311-3)
Supplement: Supplementary file 1 — Additional file 1. Additional Material and Methods. [file 13229_2019_311_MOESM1_ESM.docx]

**Additional File 1**

**Additional Materials and Methods**

**Dose response testing**

All assays were carried out using the CellTiter-Glo viability kit (Promega) according to the manufacturer’s instructions. Briefly, NPCs were seeded 24h prior to treatment in a 384-well plate format at 2x103 cells/well. For viability assays, NPCs were then treated with rapamycin or DMSO vehicle control for 72h, in triplicate, in full growth conditions. See figure legend for drug concentration. Dose response curves were generated using GraphPad Prism 8.

**Cell cycle analysis**

Cells were harvested and fixed in 4% paraformaldehyde for 20 min, stained with 20 μg/ml of propidium iodide (PI) in PBS with 1mg/ml of Rnase and incubated 15 minutes at room temperature. Samples were collected on SORP 8 laser LSR II with BD FacsDiva software. Analysis was performed with FlowJo software version 8.8.6. PI doublets were gated out using PI Area vs PI Height and analyzed with Cell Cycle Platform in FlowJo.
