# Supplementary material for: TSC patient-derived isogenic neural progenitor cells reveal altered early neurodevelopmental phenotypes and rapamycin-induced MNK-eIF4E signaling
Source: Mol Autism. 2020 Jan 6;11:2. doi: 10.1186/s13229-019-0311-3 (PMC6945400; doi:10.1186/s13229-019-0311-3)
Supplement: Supplementary file 2 — Additional file 2: Table S1. List of antibodies used. [file 13229_2019_311_MOESM2_ESM.docx]

**Additional File 2**

**Additional table S1: List of antibodies used**

| **Antibody** | **Isotype** | **Dilution** | **Manufacturer** |
| --- | --- | --- | --- |
| OCT4 | Monoclonal Mouse | 1:500 | Santa Cruz #sc5279 |
| NANOG | Polyclonal Rabbit | 1:400 | Abcam #ab80892 |
| SOX2 | Monoclonal Mouse | 1:2000 | R&D Systems #MAP2018-SP |
| MAP2 | Polyclonal Chicken | 1:2500 | EnCoR #CPCA-MAP2 |
| PHALLOIDIN | Alexa Fluor 594 | 1:5000 | ThermoFisher #A12381 |
| NESTIN | Polyclonal Rabbit | 1:500 | EMD Millipore #ABD69 |
| ERK1/2 | Polyclonal Rabbit | 1:2000 | Cell Signaling #9102 |
| p-ERK1/2 | Polyclonal Rabbit | 1:2000 | Cell Signaling #9101 |
| eIF4E | Polyclonal Rabbit | 1:1000 | Cell Signaling #9742 |
| p-eIF4E (S209) | Polyclonal Rabbit | 1:300 | Cell Signaling #9741 |
| S6 | Monoclonal Rabbit | 1:1000 | Cell Signaling #2217 |
| p-S6 | Polyclonal Rabbit | 1:1000 | Cell Signaling #2215 |
| TSC1 | Polyclonal Rabbit | 1:1000 | Cell Signaling #4906 |
| TSC2 | Polyclonal Rabbit | 1:2000 | Santa Cruz #sc893 |
| AKT | Polyclonal Rabbit | 1:2000 | Cell Signaling #9272 |
| p-AKT (S473) | Polyclonal Rabbit | 1:1000 | Cell Signaling #9271 |
| p-S6K (T389) | Monoclonal Rabbit | 1:1000 | Cell Signaling #9234 |
| TRA-1-60 MAC Sorting | Stemgent kit # 130-095-816 | | |
| PSA-NCAM MACS sorting | Stemgent kit #130-097-859 | | |
| CD271 | Miltenyi #130-099-023 | | |
| CD133 | Miltenyi #130-097-049 | | |
