# Supplementary material for: TSC patient-derived isogenic neural progenitor cells reveal altered early neurodevelopmental phenotypes and rapamycin-induced MNK-eIF4E signaling
Source: Mol Autism. 2020 Jan 6;11:2. doi: 10.1186/s13229-019-0311-3 (PMC6945400; doi:10.1186/s13229-019-0311-3)
Supplement: Supplementary file 3 — Additional file 3: Table S2. List of sgRNA or PCR primers used. [file 13229_2019_311_MOESM3_ESM.docx]

**Additional File 3**

**Additional table S2: List of sgRNA or PCR primers used**

| **sgRNA or PCR primers** | **Sequence (5’ to 3’)** |
| --- | --- |
| TSC1 Sg S (exon7) | CACCGGAGATAGACTTCCGCCACG |
| TSC1 Sg AS (exon 7) | AAACCGTGGCGGAAGTCTATCTCC |
| TSC1 genotyping ex7 Fw | CACTTGTGCTGCAACTTTCTC |
| TSC1 genotyping ex7 Rv | ATAAGGGTGTCTGGTGTGTTC |
| TSC1 genotyping ex 15 Fw | CTGGACAGACTGATACAGCAGG |
| TSC1 genotyping ex 15 Rv | CATCTGACAAACAGCAGAGAACCAG |
| TSC1 Sg 503 S (exon 15) | CACCGGGGAGACTGTCTCAGTAAA |
| TSC1 Sg 503 AS (exon 15) | TGGGAGACTGTCTCAGTAAAAGG |
| TSC1 donor sequence | AGAGGCAGAGCCTGTGGTTCCTCGAGGAGGCTTTGACTCTCCCTTTTACtGAGACAGTCTCCCAGGTTCTCAGCGGAAGACCCACTCGGCAGCCTCCAGTT |
| qPCR TSC1 Fw | CTGGACAGACTGATACAGCAGG |
| qPCR TSC1 Rv | TGCGGATCTCATCTGAAGGAGG |
| qPCR TSC2 Fw | GCACCTCTACAGGAACTTTGCC |
| qPCR TSC2 Rv | GCACCTGATGAACCACATGGCT |
| qPCR GAPDH Fw | TCGGAGTCAACGGATTTGGT |
| qPCR GAPDH Rv | TTGCCATGGGTGGAATCATA |
| qPCR Nanog Fw | ACAACTGGCCGAAGAATAGCA |
| qPCR Nanog Rv | GGTTCCCAGTCGGGTTCAC |
| qPCR Oct4 Fw | ACTGCAGCAGATGACGGAGATCG |
| qPCR Oct4 Rv | ATCCTCTCGTTGTGCATAGTCGC |
