# Supplementary figures and images for: TSC patient-derived isogenic neural progenitor cells reveal altered early neurodevelopmental phenotypes and rapamycin-induced MNK-eIF4E signaling
Source: Mol Autism. 2020 Jan 6;11:2. doi: 10.1186/s13229-019-0311-3 (PMC6945400; doi:10.1186/s13229-019-0311-3)

Additional Figures

Additional Figure 1

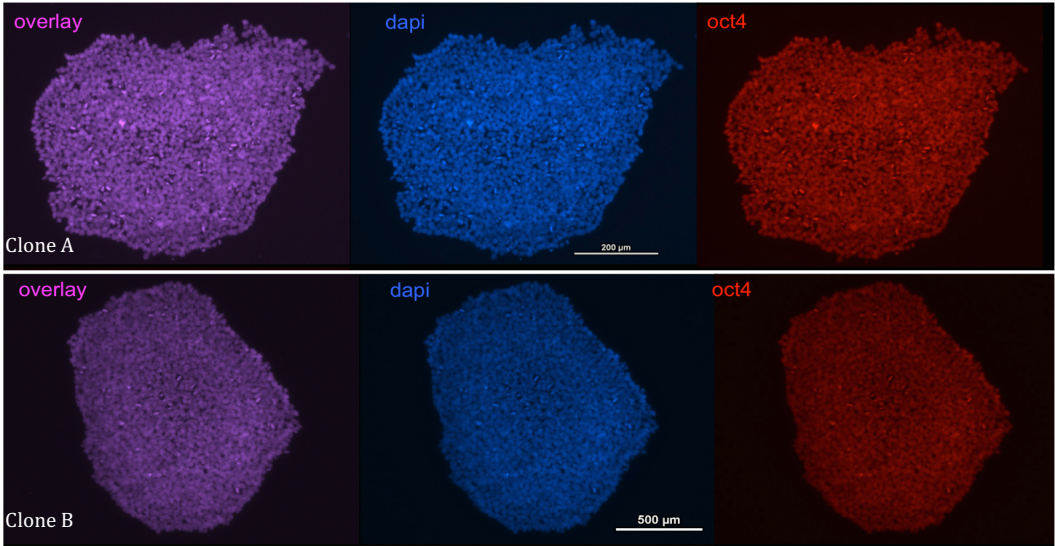

## Additional Figure 2

**A**

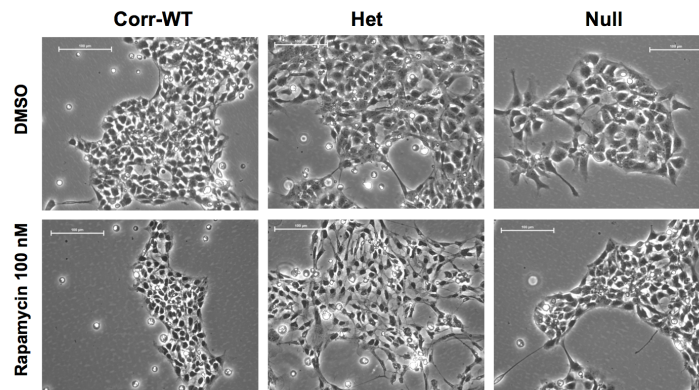

**B**

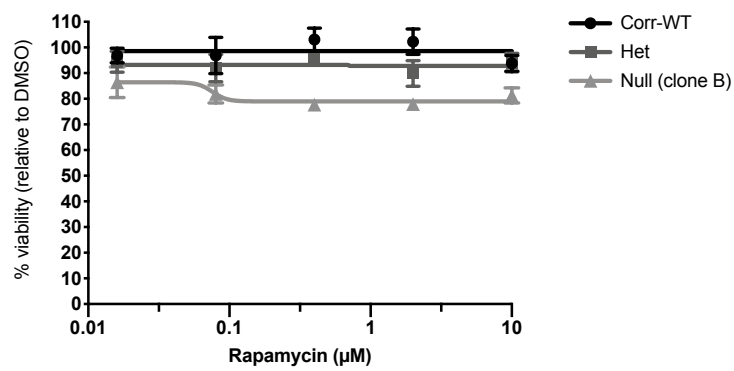

**C**

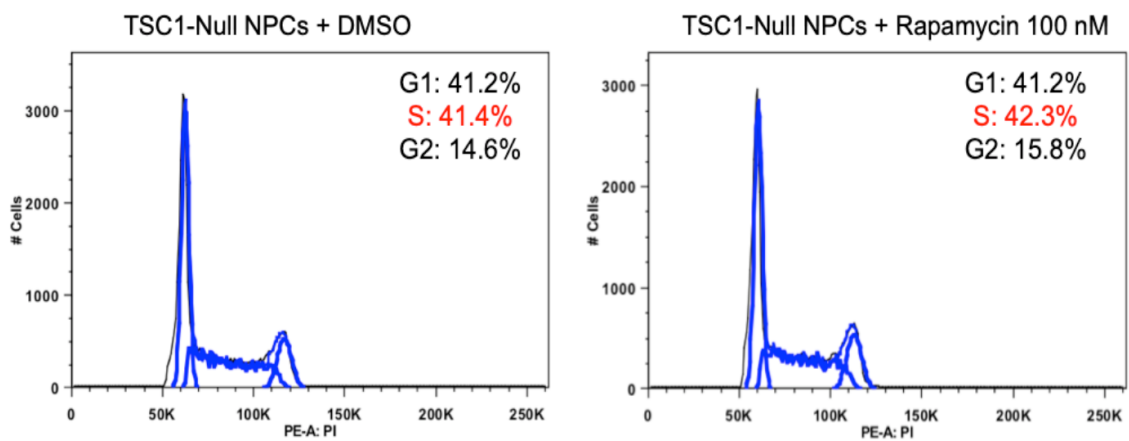

Supplement: Supplementary file 4 — Additional file 4: Figure S1. Both TSC1-Null iPSC clones (A and B) are pluripotent. Immunostaining of TSC1-Null iPSC clones after introducing a second somatic mutation in the TSC1 gene. DAPI in blue, OCT4 in red. Scale bar=500μm. Figure S2. Rapamycin reversed TSC1 NPC cell size but not proliferation. A. Bright field images of TSC1-Het and Null NPCs showing a decreased cell size after 24h treatment with 500 nM of rapamycin. Scale bar=100μm; n=3. B. TSC1 NPCs (Corr-WT, Het and Null-clone B) were treated with increasing concentrations of rapamycin in a 6 point, 5-fold serial dilution series (0 - 10 μM) for 72h. Cell viabilities were assessed using CellTiter-Glo assays and plotted as % viability (relative to DMSO). Dose response curve data is presented as +SEM (3 replicates/dose). C. TSC1-Null NPCs were treated with DMSO (left pannel) or 100 nM of rapamycin for 24h (right pannel) and stained with propidium iodide for cell cycle analysis. Proliferating cells are represented in the S phase of the cell cycle (red). [file 13229_2019_311_MOESM4_ESM.pdf]
